# Effects of an Artificial Intelligence Platform for Behavioral Interventions on Depression and Anxiety Symptoms: Randomized Clinical Trial

**DOI:** 10.2196/46781

**Published:** 2023-07-10

**Authors:** Shiri Sadeh-Sharvit, T Del Camp, Sarah E Horton, Jacob D Hefner, Jennifer M Berry, Eyal Grossman, Steven D Hollon

**Affiliations:** 1 Eleos Health Waltham, MA United States; 2 Palo Alto University Palo Alto, CA United States; 3 Ozark Center Freeman Health System Joplin, MO United States; 4 Vanderbilt University Nashville, TN United States

**Keywords:** augmentation, anxiety, artificial intelligence, cognitive-behavioral therapy, community-based center, depression, evidence-based practices, health force burnout, depressive

## Abstract

**Background:**

The need for scalable delivery of mental health care services that are efficient and effective is now a major public health priority. Artificial intelligence (AI) tools have the potential to improve behavioral health care services by helping clinicians collect objective data on patients’ progress, streamline their workflow, and automate administrative tasks.

**Objective:**

The aim of this study was to determine the feasibility, acceptability, and preliminary efficacy of an AI platform for behavioral health in facilitating better clinical outcomes for patients receiving outpatient therapy.

**Methods:**

The study was conducted at a community-based clinic in the United States. Participants were 47 adults referred for outpatient, individual cognitive behavioral therapy for a main diagnosis of a depressive or anxiety disorder. The platform provided by Eleos Health was compared to a treatment-as-usual (TAU) approach during the first 2 months of therapy. This AI platform summarizes and transcribes the therapy session, provides feedback to therapists on the use of evidence-based practices, and integrates these data with routine standardized questionnaires completed by patients. The information is also used to draft the session’s progress note. Patients were randomized to receive either therapy provided with the support of an AI platform developed by Eleos Health or TAU at the same clinic. Data analysis was carried out based on an intention-to-treat approach from December 2022 to January 2023. The primary outcomes included the feasibility and acceptability of the AI platform. Secondary outcomes included changes in depression (Patient Health Questionnaire-9) and anxiety (Generalized Anxiety Disorder-7) scores as well as treatment attendance, satisfaction, and perceived helpfulness.

**Results:**

A total of 72 patients were approached, of whom 47 (67%) agreed to participate. Participants were adults (34/47, 72% women and 13/47, 28% men; mean age 30.64, SD 11.02 years), with 23 randomized to the AI platform group, and 24 to TAU. Participants in the AI group attended, on average, 67% (mean 5.24, SD 2.31) more sessions compared to those in TAU (mean 3.14, SD 1.99). Depression and anxiety symptoms were reduced by 34% and 29% in the AI platform group versus 20% and 8% for TAU, respectively, with large effect sizes for the therapy delivered with the support of the AI platform. No group difference was found in 2-month treatment satisfaction and perceived helpfulness. Further, therapists using the AI platform submitted their progress notes, on average, 55 hours earlier than therapists in the TAU group (*t*=–0.73; *P*<.001).

**Conclusions:**

In this randomized controlled trial, therapy provided with the support of Eleos Health demonstrated superior depression and anxiety outcomes as well as patient retention, compared with TAU. These findings suggest that complementing the mental health services provided in community-based clinics with an AI platform specializing in behavioral treatment was more effective in reducing key symptoms than standard therapy.

**Trial Registration:**

ClinicalTrials.gov NCT05745103; https://classic.clinicaltrials.gov/ct2/show/NCT05745103

## Introduction

The need for scalable, empirically supported, and effective mental health care delivery approaches is a major public health priority [1]. Although many psychosocial interventions have received robust empirical support, widespread implementation in the field is still a great challenge [2,3]. Mental health centers are inundated by referrals and increased distress in the communities they support [4]. Additionally, the therapists in community-based centers face unprecedented documentation and administrative burden [5,6], leading to frontline mental health workers’ work-life imbalance and compassion fatigue [7,8]. These circumstances may impede therapists’ ability to implement evidence-based practices (EBPs) systematically and effectively [9]. Further, although most EBPs were developed and studied in academic settings, research suggests that their dissemination in community settings requires guidance on how to maintain the course when clients struggle with EBP content [10,11]. When clients do not respond to treatment, therapists may need support to lean into flexible, innovative ways of delivering, teaching, or presenting EBPs to promote engagement, understanding, and fit of therapy [12]. This reality makes it an opportune time to introduce digital tools like artificial intelligence (AI) platforms that can support therapists as they provide EBPs and seek to reduce workload [13].

Human connection, empathy, and attention to nuances are key for any effective therapy. AI-based tools can complement therapy and support providers through AI augmentation, for example, allowing them to incorporate the insights provided by AI into a client's treatment plan or delegate simple therapy-related tasks to AI automation technologies [14]. AI is capable of processing numerous data points simultaneously and does not experience burnout. AI leverages logic and can make predictions based on experience or inputs, and therefore, it can serve as an important tool for clinicians, helping to make sense of treatment data, support clinical decisions, and reduce administrative burden [15]. However, to date, there has not been a widespread implementation of AI-powered digital decision support systems and tools for operational efficiency in mental health care [16].

The goal of this randomized controlled trial (RCT), which is registered with ClinicalTrials registry (NCT05745103), was to test whether an AI platform designed to support clinical decision-making and administrative tasks in behavioral health care would be feasible and acceptable to patients and therapists. A secondary aim was to test whether using the AI platform could enhance outcomes with respect to depression and anxiety in adults receiving outpatient cognitive behavioral therapy (CBT) in a community-based clinic compared to patients receiving treatment as usual (TAU). The results of this RCT will indicate whether AI augmentation in behavioral health care leads to optimizing health outcomes for clients.

## Methods

### Recruitment

This report follows the protocol of Consolidated Standards of Reporting Trials involving AI (CONSORT-AI) [17]. All participants signed an informed consent form in person prior to participation.

### Ethics Approval

The RCT protocol was reviewed and approved by the Ozark Center’s Research Committee, the Freeman Health System Institutional Research Board, and the State of Missouri Department of Mental Health under the research title “Optimizing behavioral healthcare delivery through technology.”

### Participants

Participants in this study were 47 adults (34, 72% women and 13, 28% men; mean age 30.64, SD 11.02 years) referred to individual, outpatient CBT with a diagnosis of a depressive or anxiety disorder and the clinicians treating them. Exclusion criteria included having a severe physical or mental health condition that might interfere with treatment attendance or compliance. A total of 23 participants were randomized to the AI platform group and 24 to TAU. Most participants were White (44/47, 94%), and 6% (3/47) were Hispanic or Latino. Table 1 details participants’ demographic characteristics.

**Table 1:** Demographic characteristics of included participants.

Characteristic		AI^a^ group (n=23)	TAU^b^ group (n=24)	Total sample (n=47)
Age (years), mean (SD)		28.22 (9.46)	32.96 (12.07)	30.64 (11.02)
**Sex, n (%)**				
	Women	16 (70)	18 (75)	34 (72)
	Men	7 (30)	6 (25)	13 (28)
**Race**				
	Black or African	1 (4)	2 (8)	3 (6)
	White or European	22 (96)	22 (92)	44 (94)
**Ethnicity**				
	Hispanic or Latino	1 (4)	2 (8)	3 (6)
	Not Hispanic or Latino	22 (96)	22 (92)	44 (94)
**Education**				
	Higher education	7 (30)	9 (38)	16 (34)
	12th grade	13 (57)	13 (54)	26 (55)
	Ninth-12th grade	3 (13)	2 (8)	5 (11)
**Employment status**				
	Full-time	10 (43)	14 (58)	24 (51)
	Part-time	5 (22)	4 (17)	9 (19)
	Unemployed	8 (35)	2 (8)	10 (21)

^a^AI: artificial intelligence.

^b^TAU: treatment as usual.

### Procedure and Measures

**Figure 1:**
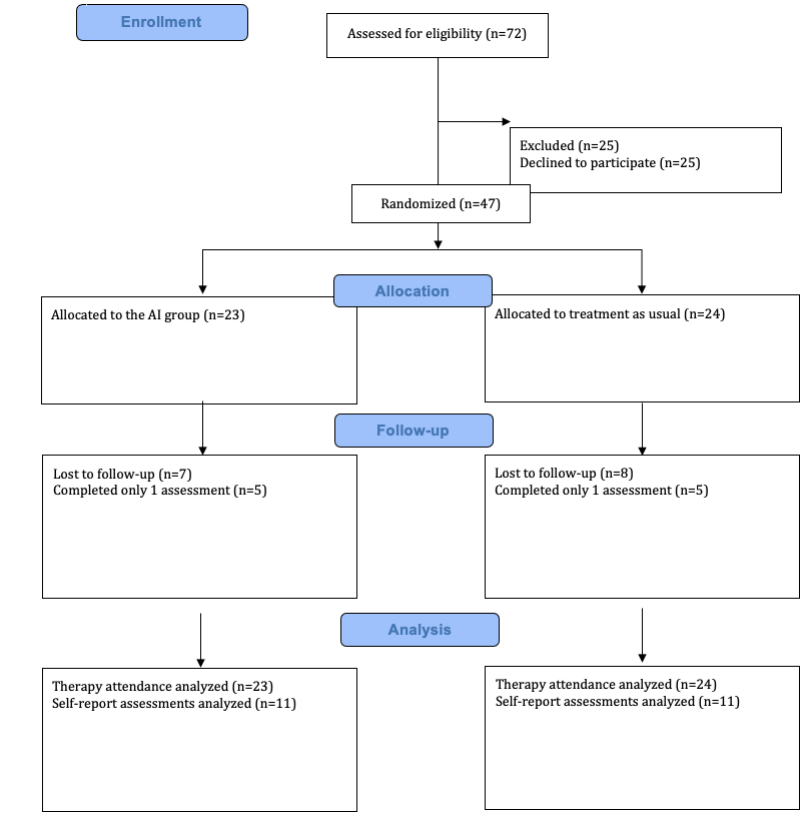
The study’s CONSORT-AI flow diagram. CONSORT-AI: Consolidated Standards of Reporting Trials-artificial intelligence.

This study was conducted at the adult outpatient program at the Ozark Center, Missouri, United States. Potential participants were initially identified in person by the center's intake team and were randomized at the therapist level to either the Eleos or TAU group after consent. Therapists in both AI and TAU arms were employees of the clinic, all master’s degree–level social workers or counselors. The primary outcomes included the feasibility and acceptability of the AI platform. Secondary outcomes included changes in depression (Patient Health Questionnaire-9; PHQ-9) [18] and anxiety (Generalized Anxiety Disorder-7; GAD-7) [19]—scores as well as treatment attendance, satisfaction, and perceived helpfulness. Participants completed depression and anxiety assessments digitally at baseline, Month 1, and Month 2. Treatment satisfaction and helpfulness were assessed at the end of the trial using 2 items rated on a 1-5 Likert scale. Qualitative feedback from therapists in the AI group was collected at the end of the trial. All assessments were completed digitally. Data on session attendance were provided by the clinic. Figure 1 presents a CONSORT-AI flowchart of participant recruitment.

### Interventions

#### The Eleos Health Platform

**Figure 2:**
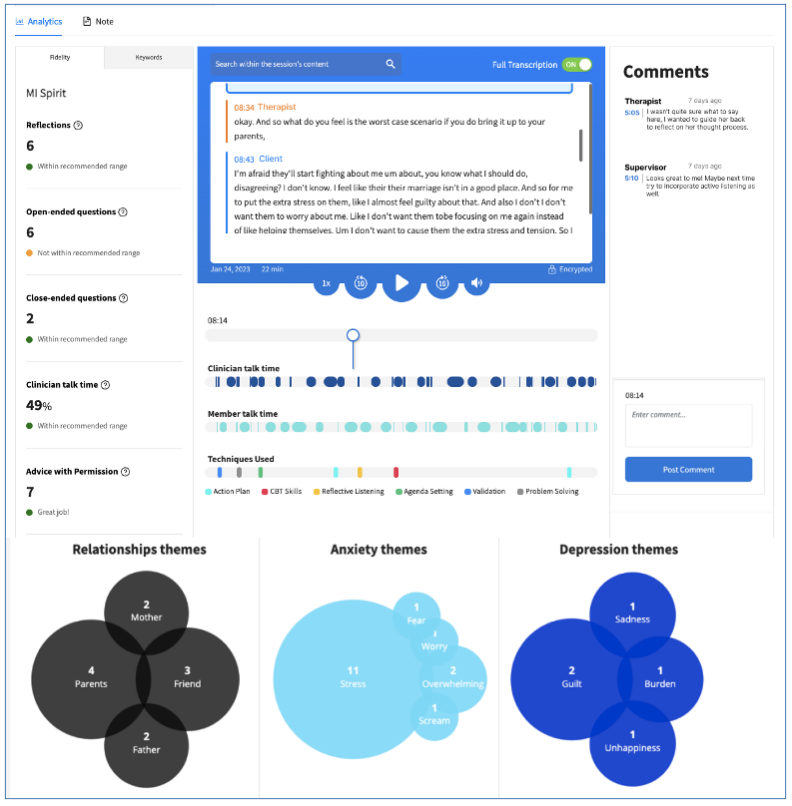
The session analytics provided by the Eleos Health platform.

**Figure 3:**
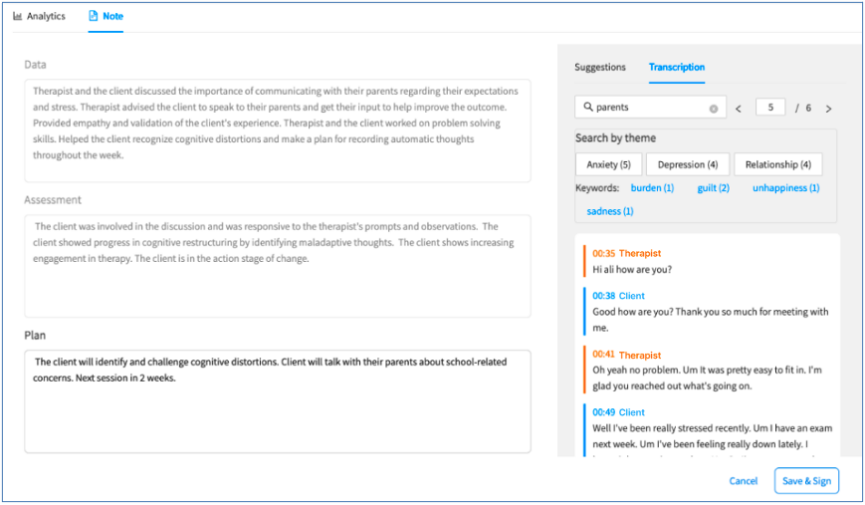
The automated progress notes provided by the Eleos Health platform the notes feature.

Therapists in the AI group used the Health Insurance Portability and Accountability Act–compliant, secure, password-protected Eleos Health platform. This AI tool was designed for behavioral health to support clinical decision-making and automation of administrative tasks. The platform captures the therapist and patient’s utterances during a treatment session, analyzes the data, and offers feedback on the implementation of EBPs (Figure 2 and [20]). The platform also incorporates a measurement-based care component, wherein standardized assessment scales completed by clients are immediately summarized and graphed for the therapist, who can then use these data to inform therapy and share them with the patient [21]. Insights and key indicators from the session data and measurement-based care are summarized into a progress note draft, which the therapist can then submit or edit as needed (Figure 3). These data can also be used in supervision, when the platform can immediately access the relevant sections of the session transcript, reducing the need to either depend upon the memory of the therapist or to listen to the entire session to find the relevant sections [22].

Therapists in the Eleos group received a 45-minute training on the platform and did not receive any additional guidance from the researchers on the interventions they should provide. Therapists were free to deliver the treatments they deemed as most effective without any specific practices prescribed.

#### TAU Group

Participants randomized to the control group received the routine services provided in the center. Therapists providing TAU were permitted to use the strategies they thought would be most successful. Therapists in both groups maintained their routine supervision and peer counseling meetings throughout the study.

### Statistical Analysis

Power analysis was based on the secondary hypothesis testing (ie, determining the effects of Eleos versus TAU on PHQ-9 and GAD-7 at the 2-month assessment). Based on pilot data [1], we anticipated a large effect size (Cohen *d*=0.8) at the 2-month assessment for both depression and anxiety. A minimum of 23 individuals were necessary for each intervention arm, according to a 2-sided test with a *P* value of .05. Baseline group differences at randomization were assessed with independent, 2-tailed *t* tests. To assess changes in depressive and anxiety symptoms over the first 2 months of therapy, we used mixed effects models for each dependent variable. In both models, the fixed explanatory variables were intervention arm and baseline values. The models also contained random effects for time. Mixed effects modeling was performed by intent-to-treat analysis using all participants who provided more than one data point (eg, [23]). Missing data points were treated as missing at random [24]. All statistical analyses were performed on SPSS statistical software (version 27; IBM Corp).

## Results

### Sample Demographics

A total of 72 adults who had been recommended outpatient CBT for a main diagnosis of a depressive or anxiety disorder were offered to participate in this study, of whom 47 (65%) provided their consent. In each group, 16 participants provided their clinical outcomes (70% and 67% of the 23 and 24 Eleos and TAU group participants, respectively). A total of 6 therapists participated in the AI group, and they treated between 1 to 6 clients; 8 therapists provided interventions in the TAU group, treating between 1 to 4 clients. Therapists could choose which study arm they wanted to join, and there were no significant group differences in the therapists’ profession, years of practice and training, or licensing status.

### Session Attendance

Participants in the AI group attended, on average, 67% more sessions than the TAU group, that is, 5.24 (SD 2.31, range 1-9) sessions, compared to 3.14 (SD 1.99, range 1-8) sessions during the first 2 months of therapy. All meetings were held in person.

### Treatment Outcomes

**Figure 4:**
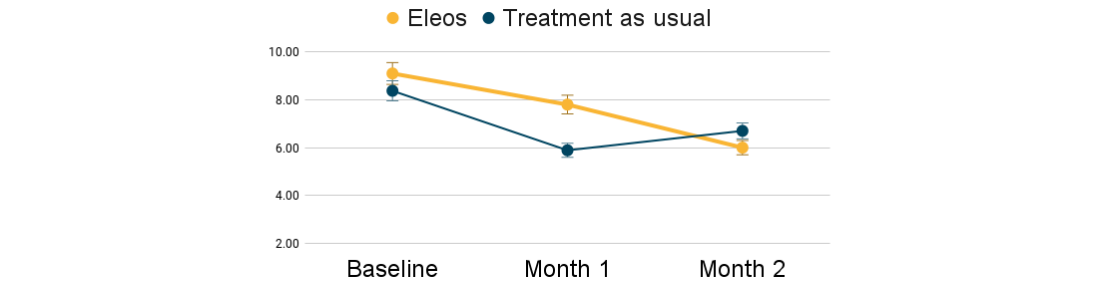
Depressive symptom change over the first 2 months of therapy. PHQ-9: Patient Health Questionnaire-9.

**Figure 5:**
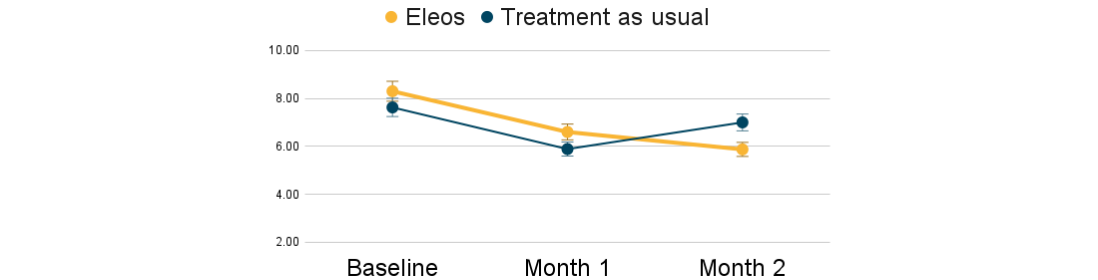
Anxiety symptom change over the first 2 months of therapy. GAD-7: Generalized Anxiety Disorder-7.

Baseline PHQ-9 scores ranged between 3 and 16, and baseline GAD-7 scores ranged between 3 and 14. No significant group differences were found at baseline in depression (*t*=0.34; *P*=.86) and anxiety symptoms (*t*=0.37; *P*=.60). Patient-reported outcomes are outlined in Table 2. Patients in both groups endorsed reductions in depression and anxiety symptoms; however, a greater symptom reduction was found among patients whose therapist used the AI platform (Figures 4 and 5). Depression symptoms were reduced by 34% with Eleos, compared to 20% for TAU, with a large effect size for the AI group (*d*=0.82) versus a small effect size for the TAU group (*d*=0.34). Anxiety symptoms were reduced by 29% with Eleos, compared to 8% for TAU, with a larger effect size for Eleos (*d*=0.78) versus a small effect size for the TAU group (*d*=0.14). One participant in the TAU group was hospitalized during the study. No additional adverse events were recorded.

**Table 2:** Group differences in depression and anxiety in the first 2 months of therapy.

Variable and group		Baseline	1 Month	2 Months	Effect sizes (95% CI) for effect baseline to 2 months	Symptom reduction from baseline to 2 months (%)	Time×group interaction effect—*F* test (df1, df2)	
**Depression (PHQ-9)^a^**								2.66 (2,11)^b^
	AI^c^	9.10 (4.70)	7.80 (4.26)	6.00 (2.56)	0.82 (–0.08 to 1.66)	34		
	TAU^d^	8.38 (4.34)	5.89 (4.34)	6.70 (5.54)	0.34 (–0.52 to 1.17)	20		
**Anxiety (GAD-7)^e^**								2.89 (2,11)^b^
	AI	8.30 (3.95)	6.60 (3.78)	5.88 (1.89)	0.78 (–0.11 to 1.62)	29		
	TAU	7.63 (3.70)	5.89 (4.34)	7.00 (5.21)	0.14 (–0.70 to 0.97)	8		

^a^PHQ-9: Patient Health Questionnaire-9.

^b^Not significant.

^c^AI: artificial intelligence.

^d^TAU: treatment as usual.

^e^GAD-7: Generalized Anxiety Disorder-7.

### AI Platform Acceptance

Patients in both groups reported high satisfaction with therapy and perceived it as highly helpful for them in achieving their goals, and no significant group differences were found; the average satisfaction rating was 4.50 (SD 0.50) in the AI group and 4.43 (SD 0.73) in the TAU group (*t*=0.20; *P*=.23); the mean perceived helpfulness of therapy was 4.25 (SD 0.66) in the AI group, compared to 4.57 (SD 0.73) in the TAU group (*t*=0.83; *P*=.81). None of the therapists or clients withdrew from the study. At the conclusion of the trial, when asked about their experience of the platform, therapists in the AI group provided positive qualitative reviews of both their augmented understanding of how they practice as well as the reduced documentation time, including the following statements: “It is very interesting to see the breakdown of CBT skills and who spoke more, etc. All the data that the program collects is fascinating”; “I believe it made me more aware of my use of CBT”; “I had some success with my documentation being more efficient”; and “It allowed for more time to engage with the client rather than being on the computer working on documentation.”

### Progress Note Quality

Therapists in the AI group submitted the progress notes for their sessions after 14 (SD 38) hours, on average, while the average submission time of the progress notes for the TAU sessions was 69 (SD 73) hours. The progress notes in both groups included required documentation aspects, such as the treatment plan, EBPs used in the session, purpose of the intervention, the client’s response, their progress toward treatment plan goals, and the plan for the next session, and they had less than 2 grammatical mistakes, on average. The notes submitted by the therapists in the AI group were, on average, 263 (SD 83) words long, while the notes submitted by the therapists in the TAU group were 318 (SD 159) words long. Table 3 presents the *t* tests and effect sizes for the progress notes’ between-group differences.

**Table 3:** Group differences in progress note characteristics between therapists using an artificial intelligence (AI) platform versus using a treatment as usual (TAU). Progress note submission time was calculated from session end time.

Variable	AI, mean (SD)	TAU, mean (SD)	*t* statistic (df)	*P* value	Effect size (95% CI)
Progress note submission time (hours)	14.03 (38.29)	69.09 (73.44)	–0.73 (222)	<.001	–1.10 (–7.64 to 5.43)
Grammatical mistakes in the progress note (n)	1.67 (1.82)	1.70 (1.81)	–0.13 (222)	.90^a^	–0.02 (–0.25 to 0.22)
Progress note length (words)	263.16 (83.51)	318.05 (159.94)	–3.33 (222)	<.001	–0.51 (–14.74 to 13.73)

^a^Not significant.

## Discussion

### Principal Findings

This study is the first RCT to report the feasibility, acceptability, and efficacy of an AI-powered platform designed to improve behavioral therapy through data and therapist feedback. With community-based clinics facing longer waitlists of patients presenting with more serious mental health difficulties, this study demonstrates the potential of digital tools to improve access to evidence-based, therapist-led care [25]. The study found that patient attendance in treatment supported by the AI platform was 2 times higher, and improvement in symptoms was 3 to 4 times better in these patients, compared to patients receiving TAU. Findings also indicate high treatment satisfaction and perceived helpfulness of therapy in both the AI and TAU groups. Additionally, the therapists in the AI group submitted the progress notes for their sessions with the study participants 55 hours earlier than therapists in the TAU group (14 hours vs 69 hours, respectively), and within the 24 hours from the service delivery time, as expected in routine care. Both groups’ notes contained the required documentation aspects and had similar, low rates of grammatical errors. However, notes from the AI group were more succinct, potentially simplifying communication with other providers.

Since this study was conducted in a routine behavioral health setting and dovetailed the interventions, procedures, and practices carried out in the clinic, the findings suggest that providing ongoing and timely feedback to therapists through an AI platform improves the quality of behavioral care provided in the field, resulting in meaningful changes in both clinical outcomes (patient retention and psychiatric symptoms) and effect sizes.

The results of this RCT suggest that providing therapy-specific AI-derived insights, such as a summary of patient’s concerns described in treatment, routine outcomes, and rate of EBPs provided by the therapist can accelerate the effects of behavioral therapy provided in a community-based clinic. Therapists in both study groups worked in the same community-based clinic and received similar training and ongoing supervision. Hence, findings indicate that community therapists can leverage technology to optimize the behavioral health services they provide. Currently, there are fewer therapists available to treat an increasing number of patients with mental health problems [26,27]. This situation is just one aspect of the overall community mental health issue. Another concern is the diminishing number of supervisors who can offer supervision to therapists with provisional licenses [28]. The AI’s ability to analyze session data and provide feedback to therapists and their supervisors is particularly noteworthy, as it allows for a more efficient use of time and expertise, and the potential for more data-informed and evidence-based care. These results demonstrate the potential for AI-powered platforms to be a key tool in addressing the growing mental health crisis, by accelerating the delivery of effective, evidence-based care and reducing the burden on community-based clinics.

### Limitations

This study included 47 participants and followed clients over a 2-month period. The therapy sessions provided by the therapists in the TAU group were not captured by the AI platform, and therefore, it is impossible to assess whether these therapists used other decision-support tools to improve practice. The small sample size and the length of the study are important limitations of this trial, particularly compared to other studies involving AI or digital tools. To further clarify these results, we encourage future research to replicate this study with a larger sample size and a longer follow-up period. Of note, the study participants presented with baseline depression and anxiety symptoms that were mostly mild to moderate in severity. Thus, future research should explore the applicability and effectiveness of AI platforms in treating individuals with severe mental illnesses and symptoms. This trial also lacks detailed data on the patients’ experience in therapy, as their satisfaction was assessed by only 2 items. The validity of AI feedback as an adjunct to clinical supervision was outside the scope of this study; however, it should be tested in future research.

### Conclusions

The findings of this RCT suggest that providing therapy in behavioral health settings with the support of an AI platform was more effective than TAU. Using the AI platform led to greater session attendance and better depressive and anxiety outcomes. Results suggest that an AI platform designed to improve EBP improves client retention and treatment outcomes in real-world settings.

## Appendix

Multimedia Appendix 1 CONSORT-eHEALTH checklist (V 1.6.1).

## Abbreviations

AI: artificial intelligence
CBT: cognitive behavioral therapy
CONSORT-AI: Consolidated Standards of Reporting Trials-artificial intelligence
EBP: evidence-based practice
GAD-7: Generalized Anxiety Disorder-7
PHQ-9: Patient Health Questionnaire-9
RCT: randomized controlled trial
TAU: treatment as usual
